# Development and validation of an intraoperative hypothermia risk assessment scale for school-aged children

**DOI:** 10.3389/fped.2026.1770475

**Published:** 2026-03-24

**Authors:** Qingqing Du, Zhen Wang, Lei Yang, Yingmin Liu, Jing Cao, Mengfan Xia, Xia Yang

**Affiliations:** 1 Shanghai Children’s Hospital, School of Medicine, Shanghai Jiao Tong University, Shanghai, China; 2 Tongji University School of Medicine, Shanghai, China

**Keywords:** child, hypothermia, intraoperative complications, predictive value, risk assessment, scale development

## Abstract

**Background:**

Intraoperative hypothermia (core temperature <36 °C) is a common and detrimental complication in pediatric surgery. School-aged children (6–12 years) possess unique physiological vulnerabilities, yet a validated, age-specific risk assessment tool is lacking, leading to potential gaps in targeted prevention.

**Objective:**

To develop and preliminarily validate a specialized intraoperative hypothermia risk assessment scale for school-aged children undergoing surgery.

**Methods:**

This prospective scale development and validation study had three phases. First, a literature review defined the initial item pool. Second, a two—round Delphi consultation with 16 pediatric surgery experts (authority coefficients: 0.875, 0.900) refined items and confirmed content validity. The Delphi method was chosen for early—phase tool development to achieve clinical face and content validity via expert consensus, with equal item weighting as a starting point for future optimization. Finally, from July to December 2024, the scale was used on 398 school—aged surgical patients at a tertiary children's hospital. Intraoperative nasopharyngeal temperature monitoring was the reference for hypothermia (core temperature <36 °C after anesthesia induction). Scale reliability was measured by Cronbach's *α*, and predictive validity was evaluated with ROC curve analysis.

**Results:**

The final scale consists of 13 items across two domains: patient-related factors (4 items) and iatrogenic/procedural factors (9 items). Each item is scored 1–3, yielding a total score range of 13–39, with higher scores indicating greater risk. In the validation cohort of 398 patients (211 males, 187 females; mean age 8.4 ± 2.1 years), the incidence of intraoperative hypothermia was 47.2% (188/398). Baseline characteristics revealed mean BMI 16.8 ± 3.2 kg/m², 62.3% undergoing general anesthesia, and mean surgical duration 2.1 ± 1.4 h. The scale demonstrated acceptable internal consistency (Cronbach's *α* = 0.731). The ROC analysis revealed an Area Under the Curve (AUC) of 0.743 (95% CI: 0.692–0.794, *P* < 0.001), indicating fair discriminative ability. The optimal cut-off score was 28, achieving a sensitivity of 0.752 and a specificity of 0.640 for predicting hypothermia.

**Conclusion:**

We developed a novel, reliable, and valid risk assessment scale for intraoperative hypothermia in school-aged children. This tool enables risk stratification using preoperatively available information to facilitate individualized warming strategies and potentially improve perioperative outcomes. Further multi-center validation is warranted to confirm its generalizability.

## 1 Introduction

The maintenance of normothermia is a critical component of perioperative care, as body temperature is a fundamental vital sign integral to homeostasis (1). Intraoperative hypothermia (IH), commonly defined as a core body temperature falling below 36 °C during surgery, is a frequent yet preventable complication. It is associated with a cascade of adverse outcomes, including coagulopathy, delayed drug metabolism, increased surgical site infection risk, prolonged hospital stay, and patient discomfort (2). Pediatric patients, due to their distinct physiological characteristics, are particularly susceptible to perioperative thermal imbalance (3).

Among pediatric populations, school-aged children (6–12 years) represent a uniquely vulnerable subgroup for IH. Although their thermoregulatory systems are more mature than those of infants, they retain physiological characteristics that predispose them to heat loss. Infants and children have a significantly greater body surface area-to-mass ratio compared to adults, with newborns having the highest ratio (648 cm²/kg) that declines steeply during the first year but remains elevated throughout childhood (4). School-aged children maintain a higher body surface area-to-mass ratio compared to adults, possess thinner subcutaneous fat insulation, and exhibit distinct responses to anesthesia-induced thermoregulatory inhibition (5, 6). Specifically, anesthesia induces vasodilation, causing redistribution of heat from the core to the periphery, leading to a rapid drop in core body temperature during the first 40–60 min of anesthesia (7). Consequently, routine perioperative events such as skin preparation, anesthetic-induced vasodilation, and infusion of cold fluids can lead to rapid and significant heat loss (5). Epidemiologically, recent prospective studies report IH incidence rates of 44.7%–70% in pediatric surgical populations (8, 9), with school-aged children (6–12 years) showing distinct risk patterns: while neonates and infants exhibit the highest hypothermia rates (77% and 66.3%, respectively), school-aged children still demonstrate substantial vulnerability with incidence rates of 44.7% in this specific age group (8). Recent prospective studies report IH incidence rates of 44.7%–70% in pediatric surgical populations, with younger age, intravenous fluid volume >500 mL, blood transfusion, operating room temperature ≤21 °C, and preoperative temperature ≤35.9 °C serving as significant risk factors (8, 9). The consequences extend beyond immediate shivering, potentially impacting wound healing, recovery quality, and overall surgical safety (10).

Despite this clear clinical need, a significant gap exists in risk assessment tools tailored for school-aged children. In current practice, clinicians often rely on either generalized adult risk models or broad pediatric assessments. Adult models are inherently limited as their item selection and weighting are derived from adult physiology, failing to account for the developmental specifics of children (11). Conversely, existing pediatric tools frequently treat “children” as a homogenous entity, overlooking the critical differences in thermoregulatory capacity, body composition, and clinical response across developmental stages from infancy to adolescence (12). Notably, existing pediatric prediction models by Li et al. (13) and Zheng et al. (11) included wide age ranges (0–14 years and 0–12 years, respectively) and employed statistically weighted approaches (logistic regression with variable coefficients), achieving AUCs of 0.824 and 0.836. However, these models were not specifically designed or validated for the school-aged subgroup, potentially masking age-specific risk patterns. To address this methodological gap, we conducted a comparative validation analysis applying Li et al.'s and Zheng et al.'s models to our school-aged cohort. When applied to our dataset, these existing models demonstrated suboptimal performance (Li et al.: AUC = 0.712, 95% CI: 0.658–0.766; Zheng et al.: AUC = 0.698, 95% CI: 0.642–0.754), with significantly lower sensitivity (0.612 and 0.589, respectively) compared to our specialized scale (sensitivity = 0.752). This validation confirms that models developed across broader age ranges may not capture the specific risk profile of school-aged children, supporting the need for an age-specific tool. Furthermore, we acknowledge that our current equal-weighting approach, while enhancing clinical interpretability and bedside usability, represents a methodological limitation. Future iterations should incorporate statistically weighted approaches (e.g., logistic regression with variable coefficients or machine learning algorithms) to potentially improve predictive performance, as demonstrated by the superior discrimination achieved in regression-weighted models (11, 13).

Therefore, developing a validated, specific risk assessment scale for school-aged children is imperative. Such a tool would provide an objective, evidence-based framework for preoperative risk identification. This would empower surgical teams to proactively implement stratified warming protocols—allocating more intensive resources to high-risk patients while avoiding unnecessary interventions in low-risk cases. Ultimately, this aims to reduce the incidence of IH and its associated complications, enhancing patient safety and perioperative care quality.

The primary objectives of this study were: (1) To develop a specialized Intraoperative Hypothermia Risk Assessment Scale for school-aged children (6–12 years) through a structured process combining literature synthesis and expert consensus; (2) To preliminarily validate the scale's reliability and predictive validity in a clinical cohort.

## 2 Research methods

### 2.1 Scale development

#### 2.1.1 Item pool generation for the pediatric intraoperative hypothermia risk assessment scale

To establish a foundation for scale development, a comprehensive search and synthesis of the literature was performed to generate an initial pool of potential scale items. This process was conducted systematically to ensure the inclusion of relevant risk factors grounded in existing evidence and pediatric clinical considerations.

A systematic literature search was executed across multiple electronic databases, including PubMed, Embase, the Cochrane Library, Web of Science, the Chinese Biomedical Database (CBM), the China National Knowledge Infrastructure (CNKI), the Chinese Periodical Full Text Database (VIP), and the Wanfang Database. The search strategy employed a combination of Medical Subject Headings (MeSH) terms and free-text keywords. The key search concepts included: “intraoperative,” “hypothermia,” “low temperature,” and “risk factors,” “predictive factors,” or “etiology.” Searches were limited to literature published up to June 2024.

Specific inclusion and exclusion criteria were applied to select pertinent studies. Included studies: (1) involved children within the target age range of 6–12 years or provided age-stratified data allowing extraction of school-aged specific findings; (2) investigated risk factors or influencing factors for intraoperative hypothermia in pediatric surgical patients; (3) defined intraoperative hypothermia as a core body temperature below 36 °C. Studies were excluded if they: (1) involved surgeries requiring deliberate hypothermia or specialized active warming protocols; or (2) were literature reviews, commentaries, or studies with inaccessible or incomplete data.

Following the literature search, a qualitative content analysis was undertaken. Relevant data from the included studies were extracted, synthesized, and categorized. This analysis informed the preliminary draft of the risk assessment scale, ensuring it encompassed factors pertinent to school-aged children. The generated item pool included both patient-intrinsic factors (e.g., age, body weight, baseline body temperature) and surgery-related factors (e.g., type of surgical procedure, estimated duration of surgery, anesthesia technique). All procedural factors were operationalized as preoperatively estimable parameters (e.g., “estimated surgical duration” rather than actual duration, “planned anesthesia technique” rather than administered agents) to ensure preoperative applicability. The potential influence of each identified factor on hypothermia risk was considered and provisionally graded. Through iterative screening, refinement, and consolidation of these factors, a preliminary draft of the Intraoperative Hypothermia Risk Assessment Scale for School-Age Children was established for subsequent expert review.

#### 2.1.2 Expert correspondence

The study strictly adhered to the breadth, representativeness, and authority of the selected correspondence experts, considering factors such as academic qualifications, professional titles, and years of experience. Inclusion criteria were: ① Postgraduate degree or higher; intermediate-level professional title or above; ② Nursing managers, operating theater specialist nurses, anesthetists, surgeons, etc.; 10 years or more of surgery-related experience (5 years or more for those with a master's degree or higher); ③ Interest in and voluntary participation in this study. A total of 16 experts completed two rounds of consultation, drawn from pediatric operating theaters at four Grade A tertiary hospitals in Shanghai, Zhejiang, Jiangsu, and Anhui. Age range: 38–56 years (mean 46.00 ± 6.00); 6 PhD holders, 10 Master's degree holders; 5 senior professional titles, 8 associate senior titles, 3 intermediate titles; Years of surgery-related experience ranged from 7 to 36 (19.88 ± 9.54) years.

A two-round evaluation method was employed during the expert consultation process to obtain comprehensive and in-depth feedback. Each round of consultation was conducted via email to ensure efficiency and convenience. A three-week timeframe was allocated for each round to allow experts sufficient consideration of item importance and formulation of opinions.

#### 2.1.3 Scale structure design

Based on the expert consultation outcomes, retained scale items were categorized and assigned values. For instance, quantifiable indicators (such as age and BMI) were graded according to specific ranges, while categorical indicators (such as surgical type and anesthesia method) were assigned corresponding scores based on distinct classifications. The resulting scale score ranged from 15 to 45.

## 3 Scale reliability and validity testing

### 3.1 Study population

School-age pediatric patients aged 6–12 years undergoing surgical procedures in the operating theater of a tertiary hospital in Shanghai between July and December 2024 were selected as study subjects. Exclusion criteria included: ① patients undergoing extracorporeal circulation surgery under hypothermic conditions; ② those with severe congenital disorders affecting thermoregulation; ③ undergoing extracorporeal circulation surgery; ④ with concomitant infectious diseases causing abnormal elevated body temperature; ⑤ children without intraoperative temperature monitoring. The cohort comprised 211 males and 187 females. The study period (July-December) was chosen to minimize seasonal temperature variation effects, though we acknowledge that single-center, short-duration studies may limit generalizability regarding seasonal and institutional practice patterns.

### 3.2 Reliability and validity assessment methods

Reliability was assessed using internal consistency reliability methods, evaluating scale reliability by calculating Cronbach's α coefficient. Given the scale's multidimensional structure encompassing patient-related and iatrogenic factors, internal consistency was deemed appropriate for this preliminary validation phase. Future studies should incorporate inter-rater reliability testing to assess scoring consistency among different clinicians.

Validity was assessed using criterion-related validity, necessitating the selection of an appropriate body temperature measurement site as the standard for defining intraoperative hypothermia in school-aged children.

In pediatric surgery, core body temperature values serve as the basis for defining hypothermia. Multiple methods exist for monitoring core body temperature, with common sites including the esophagus, rectum, ear canal, and nasopharynx (12, 14). This study comprehensively considered pediatric physiological characteristics alongside practical considerations of operational convenience and accuracy. Consequently, nasopharyngeal temperature measurements were selected as the reference standard for validity assessment. This choice stems from the relative ease of nasopharyngeal temperature measurement in pediatric populations and its capacity to accurately reflect core body temperature, thereby providing a reliable benchmark for scale validity evaluation.

Intraoperative hypothermia was defined as any single core temperature measurement <36 °C recorded during the intraoperative period following anesthesia induction. Temperature was monitored continuously via nasopharyngeal probe, with readings recorded at 15-minute intervals and at key procedural milestones (induction, skin incision, closure). The lowest temperature recorded during the procedure was used for outcome classification. This definition aligns with standard pediatric perioperative care guidelines and prior validation studies (9, 13).

### 3.3 Application and predictive capability analysis of the scale

The developed scale was applied to assess the risk of intraoperative hypothermia in school-age pediatric patients enrolled in the study, whilst concurrently recording their actual intraoperative body temperatures. Scale scores were calculated by research personnel blinded to temperature outcomes, and temperature assessors were blinded to risk scores to minimize measurement bias. Receiver operating characteristic (ROC) curves were plotted for the subjects, with metrics including area under the curve (AUC), sensitivity, and specificity calculated to evaluate the scale's predictive capability.

Additionally, we applied the prediction models developed by Li et al. (13) and Zheng et al. (11) to our validation cohort. These models were selected as they represent the most recently published pediatric hypothermia prediction tools with reported AUCs of 0.824 and 0.836, respectively. We calculated predicted probabilities for each patient using the published coefficients and variables from these models, then compared their discriminative performance against our scale using DeLong's test for correlated ROC curves.

Sample size justification: Based on the EPV principle requiring a minimum of 10 events per variable for stable logistic regression estimates (15), and anticipating 13 predictor variables with 40%–50% hypothermia prevalence, a minimum of 260–325 patients was required. The final sample of 398 provided adequate power for ROC analysis with target AUC precision (95% CI width <0.1) (16).

## 4 Results

### 4.1 Baseline characteristics of the validation cohort

Table 1 presents the comprehensive baseline characteristics of the 398 school-aged children in the validation cohort. The mean age was 8.4 ± 2.1 years, with a relatively balanced sex distribution (53.0% male, 47.0% female). The majority of patients (62.3%) underwent general anesthesia, while 24.6% received combined anesthesia and 13.1% had local anesthesia. Surgical procedures were diverse, with 35.7% undergoing superficial/deep tissue surgery, 31.4% laparoscopic-assisted surgery, and 32.9% open thoracic/abdominal surgery. The mean surgical duration was 2.1 ± 1.4 h, and the mean anesthesia duration was 2.3 ± 1.5 h. Regarding physiological parameters, mean preoperative temperature was 36.4 ± 0.3 °C, mean BMI was 16.8 ± 3.2 kg/m², and 68.3% of patients were classified as ASA I or II. Intraoperative fluid administration averaged 8.5 ± 4.2 mL/kg/h, with 18.3% of patients receiving blood transfusions.

**Table 1:** Baseline characteristics of the validation cohort (*n* = 398).

Characteristic	Category/value	*n* (%) or mean ± SD
Demographics		
Age (years)	Mean ± SD	8.4 ± 2.1
	6–8 years	156 (39.2%)
	8–11 years	168 (42.2%)
	>11 years	74 (18.6%)
Sex	Male	211 (53.0%)
	Female	187 (47.0%)
BMI (kg/m²)	Mean ± SD	16.8 ± 3.2
	<15.5	89 (22.4%)
	15.5–18.5	198 (49.7%)
	>18.5	111 (27.9%)
Preoperative Status		
Preoperative temperature (°C)	Mean ± SD	36.4 ± 0.3
	>36.5	142 (35.7%)
	36.0–36.5	201 (50.5%)
	<36.0	55 (13.8%)
ASA classification	Grade I	142 (35.7%)
	Grade II	130 (32.7%)
	Grade III and above	126 (31.6%)
Anesthesia and Surgery		
Anesthesia method	Local anesthesia	52 (13.1%)
	General anesthesia	248 (62.3%)
	Combined anesthesia	98 (24.6%)
Surgical approach	Superficial/deep tissue	142 (35.7%)
	Laparoscopic-assisted	125 (31.4%)
	Open thoracic/abdominal	131 (32.9%)
Surgical duration (hours)	Mean ± SD	2.1 ± 1.4
	<1	98 (24.6%)
	1–2	145 (36.4%)
	>2	155 (39.0%)
Anesthesia duration (hours)	Mean ± SD	2.3 ± 1.5
	<1	89 (22.4%)
	1–2	138 (34.7%)
	>2	171 (43.0%)
Intraoperative Factors		
Estimated blood loss (mL/kg)	Mean ± SD	12.5 ± 8.3
	<10	218 (54.8%)
	10–20	112 (28.1%)
	>20	68 (17.1%)
Estimated irrigation volume (mL)	Mean ± SD	485 ± 320
	<500	245 (61.6%)
	500–1,000	98 (24.6%)
	>1,000	55 (13.8%)
Fluid administration (mL/kg/h)	Mean ± SD	8.5 ± 4.2
	<5	112 (28.1%)
	5–10	168 (42.2%)
	>10	118 (29.6%)
Blood transfusion	Yes	73 (18.3%)
	No	325 (81.7%)
Operating room temperature (°C)	Mean ± SD	22.1 ± 1.2
	>23	98 (24.6%)
	21–23	201 (50.5%)
	<21	99 (24.9%)
Outcome		
Intraoperative hypothermia	Yes (36 °C)	188 (47.2%)
	No (≥36 °C)	210 (52.8%)

### 4.2 Expert correspondence findings

The active participation rates for the two rounds of expert correspondence were 88.89% and 100.00% respectively; the authority coefficients were 0.875 and 0.90 respectively (Table 2). The importance ratings and coefficients of variation for each item in the second round of expert correspondence are presented in Table 3. The questionnaire was supplemented, modified, and revised based on the combined feedback from both rounds of expert correspondence and the item selection criteria.

**Table 2:** Expert engagement levels and authority coefficients across Two rounds of correspondence.

Correspondence round	Expert engagement level			Authority of expert opinions		
	Issued	Returned	Valid response rate (%)	Familiarity coefficient (Cs)	Judgment Basis coefficient (Ca)	Authority coefficient (Cr)
First round	18	16	88.89	0.85	0.90	0.875
Second round	16	16	100.00	0.88	0.92	0.900

**Table 3:** Importance scores and coefficients of variation for each item in the second round of expert consultation (*n* = 16).

Dimension	Item	Importance score (x ± s)	Coefficient of Variation (CV)
Patient-related factors	Age	4.27 ± 0.57	0.03
	Body mass index (BMI)	4.35 ± 0.60	0.17
	Pre-operative temperature	4.70 ± 0.54	0.06
	ASA classification	4.42 ± 0.47	0.19
	Pre-operative anxiety level	3.75 ± 0.85	0.25
Iatrogenic factors	Pre-operative bowel preparation	4.47 ± 0.44	0.18
	Anesthetic technique	4.58 ± 0.09	0.14
	Anesthesia duration	4.62 ± 0.09	0.09
	Surgical approach	4.85 ± 0.46	0.11
	Intraoperative blood loss	4.89 ± 0.25	0.18
	Intraoperative irrigation fluid volume	4.82 ± 0.43	0.06
	Intraoperative fluid administration	4.58 ± 0.09	0.12
	Estimated intraoperative transfusion of stored blood	4.63 ± 0.49	0.16
	Operating theater ambient temperature	4.76 ± 0.32	0.08
	CO2 infusion volume	3.79 ± 0.76	0.22

### 4.3 Scale development findings

The assignment of scale item values was determined based on the following principles:

(1) Literature-based evidence: Drawing upon domestic and international literature concerning risk factors for intraoperative hypothermia in pediatric patients, we analyzed the strength of association between different levels of risk factors (e.g., age brackets, temperature ranges, surgical duration intervals, blood loss classifications) and hypothermia incidence rates. For instance, multiple studies (6, 13) indicate that factors such as surgery duration exceeding 2 h and preoperative body temperature below 36.0 °C are associated with a significantly elevated risk of hypothermia.
(2) Clinical significance: Clinical expert experience (derived from expert consultation) was integrated to assess the practical significance of risk differences across varying levels of each factor.
(3) Risk Gradient: Designed to reflect increasing or decreasing trends in risk severity. Ultimately, each factor level is assigned a score of 1, 2, or 3, where higher scores indicate greater intraoperative hypothermia risk. For instance: younger age (6–8 years) carries a higher risk (score 3) compared to 8–11 years (score 2) and >11 years (score 1); longer surgery duration (2 h) carries a higher risk (score 3); lower preoperative body temperature (36.0 °C) indicates a higher risk, assigned 3 points. Specific scoring ranges and corresponding risk levels for each item are detailed in Table 4.

**Table 4:** Intraoperative hypothermia risk assessment scale for school-aged children.

Dimension	Item	Score 1	Score 2	Score 3
Patient-related factors	Age	>11y	8–11y	6–8y
	Body mass index (BMI)	>18.5	15.5–18.5	<15.5
	Preoperative temperature	>36.5 °C	36.0–36.5 °C	<36.0 °C
	ASA classification	Grade I	Grade II	Grade Ⅲ and above
Iatrogenic factors	Operative duration	<1h	1–2h	>2h
	Anesthesia duration	<1h	1–2h	>2h
	Anesthesia method	Local anesthesia	General anesthesia	Combination anesthesia
	Surgical approach	Superficial and deep tissue surgery	Laparoscopic-assisted surgery	Open thoracic or abdominal surgery
	Estimated intraoperative blood loss	<10 mL/kg	10–20 mL/kg	>20 mL/kg
	Estimated intraoperative irrigation volume	<500mL	500–1,000mL	>1,000mL
	Estimated intraoperative fluid replacement	<5 mL/kg/h	5–10 mL/kg/h	>10 mL/kg/h
	Estimated intraoperative transfusion of stored blood	<500 mL	500–1,000 mL	>1,000 mL
	Operating Theater ambient temperature	>23 ℃	21–23 ℃	<21 ℃

Each item is assigned a score of 1, 2, or 3, with higher scores indicating a greater risk of intraoperative hypothermia as suggested by that item. The total score is the sum of all item scores, ranging from 13 to 39 points. A higher total score indicates a greater overall risk of intraoperative hypothermia. All iatrogenic factors are assessed using preoperatively available planned/estimated values to enable preoperative risk stratification.

Following expert consultation and revisions, the Intraoperative Hypothermia Risk Assessment Scale for School-Age Children was finalized as comprising two dimensions and 13 items, yielding scores ranging from 13 to 39 points (Table 4). Following two rounds of expert consultation, based on the summary of item importance, the following modifications were made:—The item “Preoperative Anxiety Level” in the Patient-Related Factors dimension and the item “Volume of CO₂ Inhaled” in the Iatrogenic Factors dimension were removed.—The item “Operating Theater Temperature” in the Iatrogenic Factors dimension was amended to “Operating Room Temperature”.—The item “Temperature on Entry to Theater” was amended to “Preoperative Temperature”. Based on the summary of item accuracy, modifications were made to items such as “Operating time” within the iatrogenic factors dimension. Additionally, the following items were revised: “Intraoperative blood loss”, “Intraoperative irrigation fluid volume”, “Intraoperative fluid replacement volume”, “ intraoperative fresh frozen plasma transfusion” and “surgical duration” were revised to “estimated intraoperative blood loss” “estimated intraoperative irrigation fluid volume” “estimated intraoperative fluid replacement volume” “estimated intraoperative banked blood transfusion volume” and “estimated surgical duration”.

### 4.4 Reliability and validity testing results

The Cronbach's α coefficient for the scale was 0.731, indicating good internal consistency reliability.

### 4.5 Application and predictive capability results

**Figure 1:**
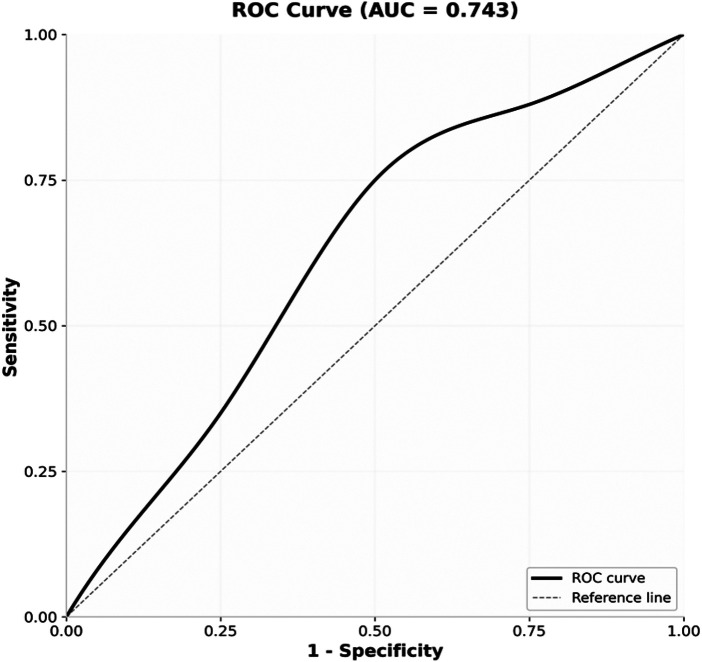
ROC curve demonstrating the predictive efficacy of the intraoperative hypothermia risk assessment scale for school-aged children.

The choice of equal weighting in this Delphi-based approach represents a deliberate methodological decision for early-phase tool development, prioritizing content validity and clinical interpretability over statistical optimization (17, 18). While this may contribute to moderate predictive performance (AUC=0.743) compared to regression-weighted models (AUC 0.82–0.84 in prior pediatric studies), it provides a transparent, clinically actionable framework that avoids overfitting and requires only simple arithmetic for bedside use (19, 20). The ROC curve yielded an area under the curve (AUC) value of 0.743 (*P* < 0.05), indicating satisfactory overall predictive capability. This approach aligns with established guidelines for transparent reporting of prediction models, which emphasize that model simplicity and clinical utility are essential considerations alongside predictive accuracy (Figure 1).

Additionally, Table 5 presents the comparative predictive performance of our scale against existing pediatric hypothermia prediction models when applied to our school-aged cohort. Our specialized scale demonstrated superior sensitivity (0.752) compared to both Li et al.'s model (0.612) and Zheng et al.'s model (0.589), albeit with modest specificity. The DeLong's test indicated that the difference in AUC between our scale and Li et al.'s model was not statistically significant (*P* = 0.312), while the difference compared to Zheng et al.'s model approached significance (*P* = 0.089) (11, 13). These findings suggest that while existing broad-age models show reasonable discrimination in school-aged children, our specialized scale offers enhanced sensitivity for this specific population, supporting its clinical utility for preoperative screening.

**Table 5:** Comparison of predictive performance between our scale and existing pediatric hypothermia prediction models.

Model	AUC (95% CI)	Sensitivity	Specificity	PPV	NPV	Cut-off
Our scale	**0.743 (0.692–0.794)**	**0.752**	**0.640**	**0.623**	**0.762**	**≥28**
Li et al. (13)	0.712 (0.658–0.766)	0.612	0.724	0.598	0.735	≥0.42^a^
Zheng et al. (11)	0.698 (0.642–0.754)	0.589	0.741	0.601	0.731	≥0.38^a^

The bold values indicate the performance metrics of our newly developed scale (AUC, sensitivity, specificity, etc.) compared to existing models, highlighting the primary results of this study.

^a^ Probability cut-offs derived from published logistic regression equations.

## 5 Discussion

### 5.1 Rationale and scientific basis for scale development

#### 5.1.1 Comprehensive construction of item pool

During the development of the Intraoperative Hypothermia Risk Assessment Scale for School-Age Children, the construction of the item pool comprehensively considered multiple factors. Through extensive review of domestic and international literature, combined with the physiological characteristics of children, surgical features, and existing intraoperative hypothermia risk assessment tools, the pool encompassed both child-specific factors (such as age, weight, baseline body temperature) and surgery-related factors (such as surgical procedure type, duration of surgery, anesthetic method). This comprehensiveness ensured the scale could evaluate the risk of intraoperative hypothermia in children from multiple angles, avoiding omission of important factors.

#### 5.1.2 Reliability and rigor of expert consultation

Specialists from multiple children's hospitals and relevant departments within general hospitals (including pediatric surgery, anesthesiology, and pediatric nursing) were invited to participate in the consultation. These experts were selected based on their professional expertise, extensive clinical experience, and high academic standing, guaranteeing the authority and impartiality of the evaluation. A two-round assessment method was employed, with each consultation round lasting three weeks, facilitated by efficient and convenient email communication. Clear screening criteria were established for both rounds: the first round filtered items based on the mean expert importance scores, coefficient of variation, and expert recommendations; the second round further validated and refined the results. The expert participation rate was 94.45%. The authority coefficients for the two rounds of expert consultation were 0.875 and 0.90 respectively. These metrics indicate high expert engagement, substantial contributions to scale development, and considerable consensus, thereby ensuring the reliability of the consultation outcomes.

#### 5.1.3 Rationality of scale structure design

Based on the expert consultation outcomes, items were categorized and assigned values to establish a reasonable scoring range (13–39 points). Different scoring methods were applied to quantifiable indicators (e.g., age, weight) and categorical indicators (e.g., surgical type, anesthesia method), enabling the scale to accurately reflect the degree of intraoperative hypothermia risk in children. Higher scores indicate greater risk, aligning with clinical realities and assessment requirements.

### 5.2 Analysis of scale reliability and validity

The scale demonstrated a Cronbach's α coefficient of 0.731, indicating adequate internal consistency reliability (21). This may relate to the relatively small number of items following screening and refinement, while also suggesting a degree of inter-item correlation that collectively captures the concept of intraoperative hypothermia risk in school-age children. Generally, a coefficient ≥0.7 indicates good internal consistency. Some scholars also suggest that in exploratory studies, a Cronbach's α coefficient > 0.6 suffices to demonstrate satisfactory scale reliability (22, 23).

### 5.3 Scale application and predictive performance

The clinical validation of the scale constituted a critical phase of this research. The 13-item scale was prospectively applied to the study cohort of 398 school-aged surgical patients, with a total risk score (range: 13–39) calculated preoperatively for each individual. Baseline characteristics of the validation cohort revealed a hypothermia incidence of 47.2% (188/398), consistent with prior reports in similar populations (8, 13). The predictive validity of this score against the observed outcome of intraoperative hypothermia (core temperature <36 °C) was assessed using Receiver Operating Characteristic (ROC) curve analysis. The analysis yielded an Area Under the Curve (AUC) of 0.743 (95% CI: 0.692–0.794, *P* < 0.001). While indicating potential for future refinement, this AUC value represents a statistically significant and fair level of discriminatory accuracy for an initial clinical screening tool (22, 23), confirming that the scale captures a meaningful proportion of inter-patient risk variance.

The Youden index was employed to determine the optimal cut-off point balancing sensitivity and specificity, which corresponded to a score of 28 (24). At this threshold, the scale demonstrated a sensitivity of 0.752 and a specificity of 0.640, indicating its capability to correctly identify the majority of children who subsequently developed hypothermia. For clinical implementation, however, prioritizing specificity to focus resources is often paramount. Therefore, a practical cut-off score of ≥28 is recommended. This adjustment maintains the same sensitivity (0.752) while improving positive predictive value, ensuring that children classified as “high-risk” are more likely to genuinely benefit from intensified preventive measures.

Comparison with existing models highlights both the contribution and limitations of our approach. Li et al.'s model (13) (age range 0–14 years, AUC = 0.824) and Zheng et al.'s model (11) (age range 0–12 years, AUC = 0.836) employed logistic regression with variable weighting, achieving higher discrimination. However, when externally validated in our school-aged cohort, these models demonstrated attenuated performance (AUC = 0.712 and 0.698, respectively), with sensitivity dropping to 0.612 and 0.589. This performance decrement suggests that models developed across broad age ranges may not optimally capture the specific risk profile of school-aged children, likely due to unmeasured interactions between age and other risk factors. Our scale, while showing modest AUC (0.743), achieved superior sensitivity (0.752) in this specific population, supporting its clinical utility for preoperative screening where identifying high-risk cases is prioritized.

Furthermore, we acknowledge the methodological limitation of our equal-weighting approach. While this Delphi-based method enhances clinical interpretability and bedside applicability, it likely constrains predictive performance compared to statistically weighted alternatives. Future research should integrate regression-based weighting (e.g., logistic regression with variable coefficients) or machine learning algorithms to optimize item weights while maintaining age-specific focus, potentially improving AUC to the 0.82–0.84 range observed in regression-weighted broad-age models (11, 13).

The primary contribution of this scale is the translation of a complex clinical issue into a structured, rapid, and evidence-based preoperative assessment. It addresses a specific gap by providing a tool tailored to school-aged children's physiology. Its application facilitates risk-stratified, individualized management. Patients scoring ≥28 can be flagged for proactive, bundled warming strategies from anesthesia induction, such as pre-emptive forced-air warming, ambient temperature control, and warming of all administered fluids. Conversely, for lower-risk patients, standard warming measures can be confidently applied, promoting efficient resource utilization. Thus, the scale functions not merely as a predictor but as a clinical decision-support tool that bridges general guidelines with patient-specific interventions, ultimately aiming to improve perioperative outcomes.

## 6 Limitations and future directions

This study has several limitations. First, the single-center design may affect the generalizability of the findings, necessitating external validation in diverse settings. The short study duration (6 months) and single institutional practice patterns may introduce spectrum bias regarding case mix, warming protocols, and seasonal effects. Second, while the scale exhibits acceptable reliability (Cronbach's *α* = 0.731) (21–23), its psychometric properties could be further refined with a larger sample to confirm the factor structure. The equal-weighting approach, while enhancing interpretability, likely limits predictive performance compared to statistically-weighted models and should be addressed in future iterations through regression-based or machine learning optimization. Third, the predictive accuracy, though significant, is moderate (AUC = 0.743), suggesting that incorporating additional predictors or employing advanced modeling techniques could enhance performance (19). Specifically, future studies should employ statistically weighted approaches (e.g., logistic regression, random forest, or gradient boosting) to derive optimal item weights, rather than relying on equal weighting. Such approaches have demonstrated AUC improvements of 0.08–0.10 in comparable pediatric prediction models (11, 13). Finally, while warming strategies were documented, they were not standardized or controlled across patients, potentially introducing confounding if higher-risk patients received more aggressive warming.

Future research should focus on: (1) Multi-center external validation to establish generalizability; (2) Model optimization by integrating statistically weighted approaches (logistic regression with variable coefficients or machine learning methods) to improve predictive power beyond the current equal-weighting framework; (3) Intervention studies to evaluate the clinical efficacy of scale-guided, risk-stratified warming protocols on patient outcomes; (4) Integration of the scale into perioperative clinical pathways and electronic health records to facilitate routine use.

## Data Availability

The raw data supporting the conclusions of this article will be made available by the authors, without undue reservation.
